# GPRC5D as a promising therapeutic target in EGFR-wild and immuno-cold non-small cell lung cancer

**DOI:** 10.1186/s12967-023-04415-w

**Published:** 2023-08-13

**Authors:** Jie Mei, Yun Cai, Guanyu Jiang, Zhao He, Ruixin Wang, Chenghu Song, Yuan Wan, Wenjun Mao

**Affiliations:** 1 Department of Oncology, The Affiliated Wuxi People’s Hospital of Nanjing Medical University, Wuxi Medical Center, Nanjing Medical University, Wuxi, 214023 China; 2 Department of Cardiothoracic Surgery, The Affiliated Wuxi People’s Hospital of Nanjing Medical University, Wuxi Medical Center, Nanjing Medical University, Wuxi, 214023 China; 3 The Pq Laboratory of BiomeDx/Rx, Department of Biomedical Engineering, Binghamton University, Binghamton, NY 13902 USA

## To the editor

Lung cancer (NSCLC) is one of the most widespread cancers in the world, and non-small cell lung cancer (NSCLC) accounts for more than 85% of total diagnoses [1]. Although EGFR-TKIs and immune checkpoint inhibitors (ICIs) dependent on molecular expression profiling have changed the therapeutic landscape of advanced NSCLC [2], a large proportion of patients with EGFR-wild and immuno-cold tumors still lack effective treatment strategies. In our previous research, we found that the activity of cholesterol synthesis was enhanced in EGFR-wild/immuno-cold tumors, and targeting cholesterol synthesis using statins significantly boosted immunotherapy response [3]. However, given the complexity of EGFR-wild/immuno-cold NSCLC, it is necessary to further explore novel therapeutic targets in this subtype.

GPRC5D is a member of the G protein-coupled receptor family. Although its role has not been well established, GPRC5D has been used as a star target in multiple myeloma [4]. The time of GPRC5D as a therapeutic target is short, but its excellent efficacy greatly promotes the application of GPRC5D-directed CAR-T therapy in multiple myeloma [5]. According to the latest clinical trial, the response rate of GPRC5D-directed CAR-T therapy is over 70% [6]. Thus, GPRC5D is an active immunotherapeutic target in multiple myeloma. However, whether GPRC5D could be potential therapeutic target in other tumors has not been reported.

**Fig. 1:**
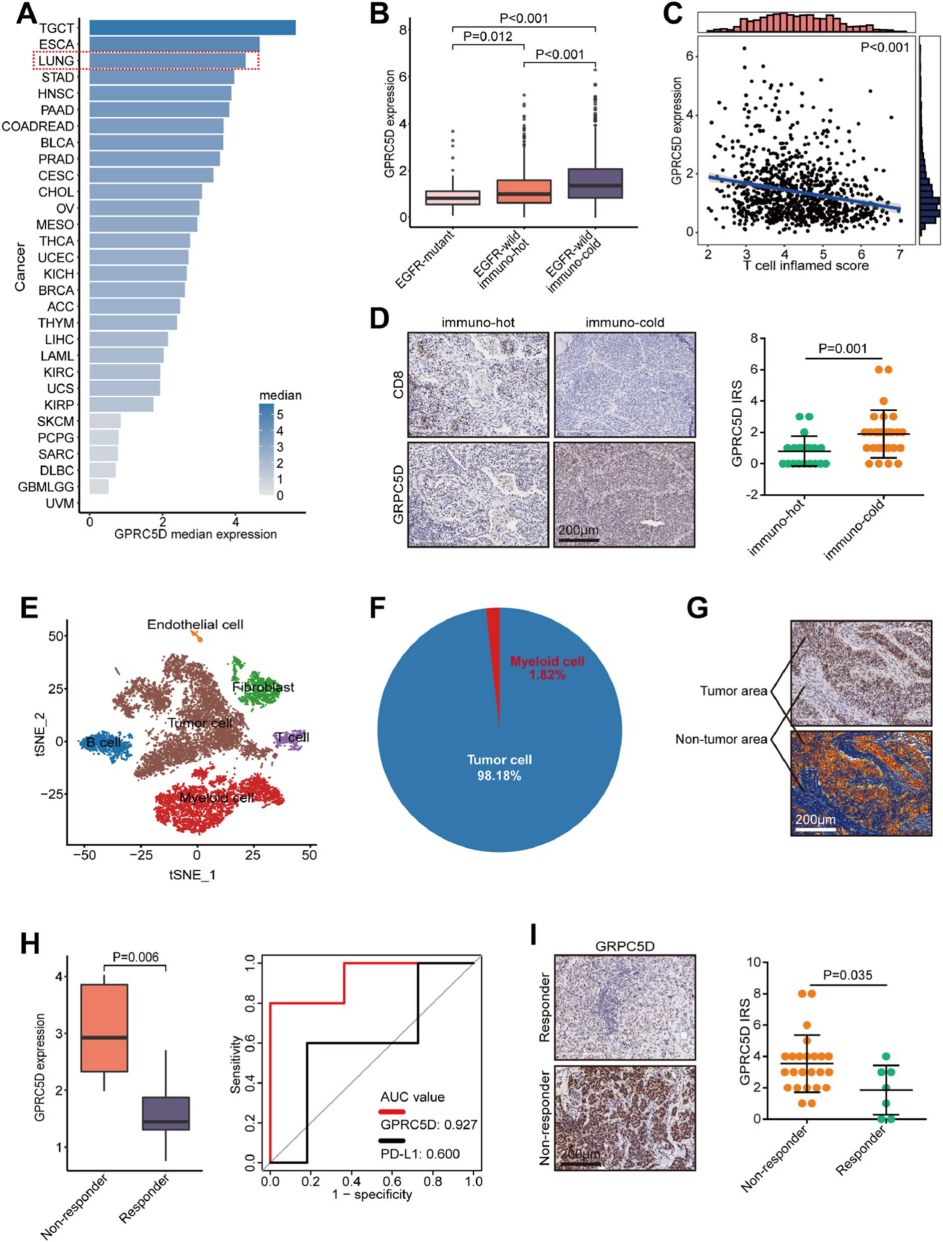
Expression of GPRC5D in NSCLC and its predictive value for immunotherapy. **A** Expression atlas of GPRC5D in pan-cancer. The data was obtained from the TCGA dataset. **B** Expression of GPRC5D in EGFR-mutant, EGFR-wild/immuno-hot, and EGFR-wild/immuno-cold tumors. The data was obtained from the TCGA dataset. **C** Correlation between GPRC5D expression and T cell inflamed score. The data was obtained from the TCGA dataset. **D** Representative images showing GPRC5D expression in EGFR-WT and immuno-hot and EGFR-WT and immuno-cold NSCLC groups, along with semi-quantitative analysis. Total original magnification, ×200. **E** The tSNE plot displaying the main cell types in the scRNA-seq dataset. **F** Distribution of GPRC5D-positive cells. **G** Expression of GPRC5D in tumor and non-tumor region. **H** Expression of GPRC5D in tumors from responders and non-responders and ROC analysis. The data was obtained from the GSE126044 dataset. **I** Representative images showing GPRC5D expression in tumors from responders and non-responders in the in-house cohort, along with semi-quantitative analysis. Total original magnification, ×200

In this report, we first conducted pan-cancer analysis to detect the expression of GPRC5D, the results showed that GPRC5D was highest expressed in NSCLC among all cancer types in addition to testicular germ cell tumor and esophageal cancer (Fig. 1A). Given the significance of EGFR status and immune subtype in NSCLC treatment, the expressions of GPRC5D in EGFR-mutant, EGFR-wild/immuno-hot, and EGFR-wild/immuno-cold tumors were compared, and it was revealed that GPRC5D was highly expressed in EGFR-wild/immuno-cold tumors (Fig. 1B). In addition, GPRC5D was negatively correlated with T cell inflamed score in EGFR-wild tumors (Fig. 1C). To further validated these findings, we examined GPRC5D expression in the previous EGFR-wild NSCLC cohort [3], and the results exhibited that GPRC5D was highly expressed in EGFR-wild/immuno-cold tumors (Fig. 1D). Single-cell expression profile analysis revealed GPRC5D was highly expressed in tumor cells (Fig. 1E, F). IHC analysis also validated that GPRC5D was highly expressed in tumor cells (Fig. 1G). In addition, compared with resectable tumors, GPRC5D was highly expressed in advanced NSCLC (Additional file 1: Fig. S1). All findings indicated GPRC5D is a promising target in NSCLC.

We further checked the correlation between GPRC5D and anti-tumor immunity. IPS score was significantly decreased in tumors with GPRC5D high expression (Additional file 1: Fig. S2A). A majority of chemokines, immunostimulators, immunoinhibitors, MHC molecules, and receptors were highly expressed in the low GPRC5D group (Additional file 1: Fig. S2B). We further checked whether GPRC5D could be a biomarker for immunotherapy in NSCLC. In the GSE126044 cohort, GPRC5D was highly expressed in tumors from non-responders, and the predictive value of GPRC5D was even higher than PD-L1 (Fig. 1H). Furthermore, an in-house immunotherapy cohort was also included to validate the above result (Additional file 1: Table S1), and the result was confirmed (Fig. 1I). However, in other cancer types suitable for immunotherapy, such as melanoma, esophageal cancer, gastric cancer, and bladder cancer, GPRC5D could not predict the immunotherapeutic responses (Additional file 1: Fig. S3).

## Conclusion

In conclusion, we revealed that GPRC5D was highly expressed in NSCLC and enhanced in the EGFR-wild and immuno-cold type. In addition, GPRC5D associated with immuno-cold features and low response to anti-PD-1/PD-L1 immunotherapy in NSCLC. Thus, targeting GPRC5D could be a novel therapeutic target in EGFR-wild and immuno-cold NSCLC, and further research should highlight the importance of GPRC5D in this type of refractory NSCLC.

### Supplementary Information

**Additional file 1.** **Figure S1**. Expression of GPRC5D in resectable and advanced tumors. **Figure S2**. Correlation between GPRC5D expression and (A) IPS and (B) immunomodulators. **Figure S3**. Expression of GPRC5D in tumors from responders and non-responders in other cancer types. **Table S1**. The baseline clinic-pathological features of two cohorts.

## Data Availability

The datasets used and/or analyzed during the current study are available from the corresponding author on reasonable request.
