# Supplementary material for: GPRC5D as a promising therapeutic target in EGFR-wild and immuno-cold non-small cell lung cancer
Source: J Transl Med. 2023 Aug 13;21:542. doi: 10.1186/s12967-023-04415-w (PMC10424388; doi:10.1186/s12967-023-04415-w)
Supplement: Supplementary file 1 — Additional file 1. Figure S1. Expression of GPRC5D in resectable and advanced tumors. Figure S2. Correlation between GPRC5D expression and (A) IPS and (B) immunomodulators. Figure S3. Expression of GPRC5D in tumors from responders and non-responders in other cancer types. Table S1. The baseline clinic-pathological features of two cohorts. [file 12967_2023_4415_MOESM1_ESM.docx]

**Additional Methods**

**Public data acquisition**

The RNA-sequencing (RNA-seq) data of NSCLC (IlluminaHiSeq, log_2_(x+1) transformed RSEM normalized count) and corresponding clinical information as well as pan-cancer RNA-seq data in The Cancer Genome Atlas (TCGA) database was downloaded from the UCSC Xena portal (http://xenabrowser.net/datapages/). Concerning the T cell inflamed score, which was calculated based on the 18 specific genes related to T cell inflammation and their weighting coefficients [1], the top 50% cases were designated as the immuno-hot subgroup, and the rest were assigned to the immuno-cold group. Finally, the EGFR-mutation group included 77 patients, the EGFR-WT & immuno-hot group included 441 patients, and the EGFR-WT & immuno-cold included 440 patients. In addition, the clinical annotation of the TCGA samples could be found in our previous research [2].

In addition, the GSE126044 data set, which contained the RNA-seq data from NSCLC patients before receiving anti–PD-1 immunotherapy, was downloaded from the Gene Expression Omnibus database (<https://www.ncbi.nlm.nih.gov/geo/>) [3].

**Description of immunological features**

The immunological characteristics of the tumor microenvironment (TME) in NSCLC mainly contained immunomodulators and immunophenoscore (IPS). The expressions of immunomodulators, including MHC, chemokines, receptors, immunoinhibitors, and immunostimulators, were regarded as the immunological characteristics of TME [4]. As previously reported, a patient’s IPS can be calculated without bias using machine learning by consideration of the 4 major categories of components that measure immunogenicity: effector cells, immunosuppressive cells, MHC molecules, and immunomodulators [1]. The IPS values of BC patients were obtained from the Cancer Immunome Atlas (TCIA) (https://tcia.at/home).

**ScRNA-seq and bioinformatics analysis**

The single-cell RNA sequencing datasets of NSCLC patients were obtained from GSE148071 [5]. All additional analyses were performed using the Seurat (4.0.4, <http://satijalab.org/seurat/>) R toolkit [6], including quality control and all subsequent analyses.

To avoid the influence of abnormal cells and experimental noise on downstream analysis, we retained the high-quality cells based on the following criteria: (1) the number of detected genes was between 200 and 5,000; (2) the percentage of reads mapped to the mitochondrial genome was less than 10%. Finally, a total of 15,382 cells were reserved for downstream analysis. To account for multiple biological and experimental characteristics, the Harmony algorithm [7] was used to integrated the 36,634 cells from NSCLC patients. The principal component analysis (PCA) was performed on the top 4,000 genes with the highest variability. Subsequently, the first 20 principal components (PCs) were used to reduce the dimensionality of the scaled integrated dataset to two-dimensional space. The cell clusters were recognized by a shared nearest neighbor (SNN) modularity optimization-based clustering algorithm with a resolution of 0.8. According to the expression levels of some well-known markers, the 15,382 cells were annotated as ten cell types, including tumor cells, endothelial cells, myeloid cells, fibroblasts, B cells and T cells.

**Collection of NSCLC specimens**

Two independent single-center cohorts were included in the current study. The first clinical cohort included 48 NSCLC patients harboring WT EGFR from 2016 to 2020 in The Affiliated Wuxi People’s Hospital of Nanjing Medical University. The paraffin-embedded tumor samples from these patients were collected for IHC staining. In addition, a total of 31 NSCLC patients receiving immuno-chemotherapy and single anti-PD-1 immunotherapy from 2019 to 2021 were recruited by The Affiliated Wuxi People’s Hospital of Nanjing Medical University. Primary or metastatic tumor tissue samples were obtained by centesis before the start of immunotherapy. The therapeutic response was evaluated according to the RECIST 1.1 criteria, which were demarcated into complete response (CR), partial response (PR), stable disease (SD), and progressive disease (PD). The baseline clinic-pathological features were exhibited in Table S1. The first cohort was resectable NSCLC and the second cohort was advanced NSCLC. Ethical approval for the recruitment of immunotherapy cohort was granted by the Clinical Research Ethics Committee in The Affiliated Wuxi People’s Hospital of Nanjing Medical University.

**Study approval**

Collection of clinical samples in this study was approved by the IRB of The Affiliated Wuxi People’s Hospital of Nanjing Medical University (no. KY21126).

**Immunohistochemistry (IHC) staining and semi-quantitative assessment**

IHC staining was conducted on the above sections according to the standardized procedures. The sections were then washed with xylene for three 5-min. The sections were rehydrated by successive washes in 100, 90 and 70% graded ethanol. Hydrogen peroxidase was used to block endogenous peroxidase activity for 20 min. The antigen retrieval solution is EDTA. The primary antibodies used were as follows: anti-GPRC5D (1:500 dilution, Cat. 21089-1-AP, ProteinTech) and anti-PD-L1 (Ready-to-use, Cat. GT2280, GeneTech). Antibody staining was visualized with DAB and hematoxylin counterstain, and stained sections were captured using Aperio Digital Pathology Slide Scanners.

The stained sections were independently evaluated by two pathologists. Expression levels of GPRC5D and PD-L1 in tumor cells were semi-quantitatively assessed according to the 12-point criterion by calculating the immunoreactivity score (IRS) [8]. Briefly, the percentage of positively stained cells was scored as 0-4: 0 (< 5%), 1 (6-25%), 2 (26-50%), 3 (51-75%) and 4 (>75%). The staining intensity was scored as 0-3: 0 (negative), 1 (weak), 2 (moderate), and 3 (strong). The IRS equals to the percentages of positive cells multiplied with staining intensity.

**Statistical analysis**

All statistical analyses were conducted using SPSS 26.0 and R 4.0.2. All data are presented as means ± SDs. The difference between the two groups was analyzed by Student’s t-test or Mann Whitney test. Correlation analysis between two variables was analyzed by the Pearson test. All statistical tests were two-sided, and P-value < 0.05 was considered statistically significant.

**References**

1. Charoentong P, Finotello F, Angelova M, Mayer C, Efremova M, Rieder D, et al. Pan-cancer Immunogenomic Analyses Reveal Genotype-Immunophenotype Relationships and Predictors of Response to Checkpoint Blockade. Cell Rep. 2017;18(1):248-62.

2. Mao W, Cai Y, Chen D, Jiang G, Xu Y, Chen R, et al. Statin shapes inflamed tumor microenvironment and enhances immune checkpoint blockade in non-small cell lung cancer. JCI Insight. 2022.

3. Cho JW, Hong MH, Ha SJ, Kim YJ, Cho BC, Lee I, et al. Genome-wide identification of differentially methylated promoters and enhancers associated with response to anti-PD-1 therapy in non-small cell lung cancer. Exp Mol Med. 2020;52(9):1550-63.

4. Mei J, Cai Y, Xu R, Zhu Y, Zhao X, Zhang Y, et al. Protocol to identify novel immunotherapy biomarkers based on transcriptomic data in human cancers. STAR Protocols. 2023;4(2):102258.

5. Wu F, Fan J, He Y, Xiong A, Yu J, Li Y, et al. Single-cell profiling of tumor heterogeneity and the microenvironment in advanced non-small cell lung cancer. Nat Commun. 2021;12(1):2540.

6. Butler A, Hoffman P, Smibert P, Papalexi E, Satija R. Integrating single-cell transcriptomic data across different conditions, technologies, and species. Nat Biotechnol. 2018;36(5):411-20.

7. Korsunsky I, Millard N, Fan J, Slowikowski K, Zhang F, Wei K, et al. Fast, sensitive and accurate integration of single-cell data with Harmony. Nat Methods. 2019;16(12):1289-96.

8. Mei J, Liu Y, Yu X, Hao L, Ma T, Zhan Q, et al. YWHAZ interacts with DAAM1 to promote cell migration in breast cancer. Cell Death Discov. 2021;7(1):221.

**Additional Figures**


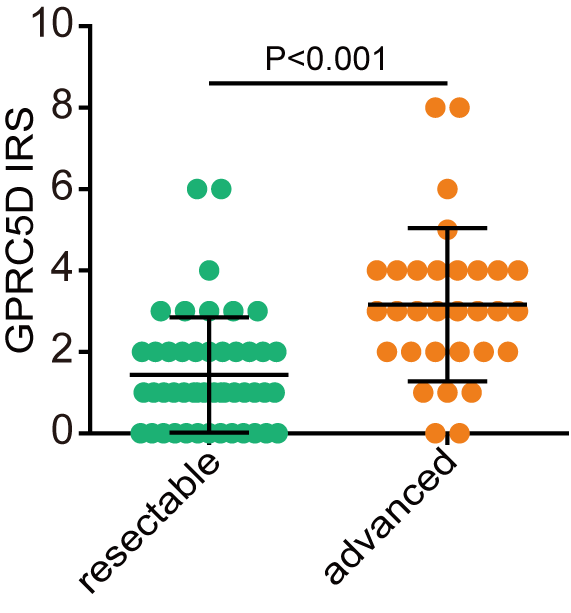


**Figure S1. Expression of GPRC5D in resectable and advanced tumors.**

**
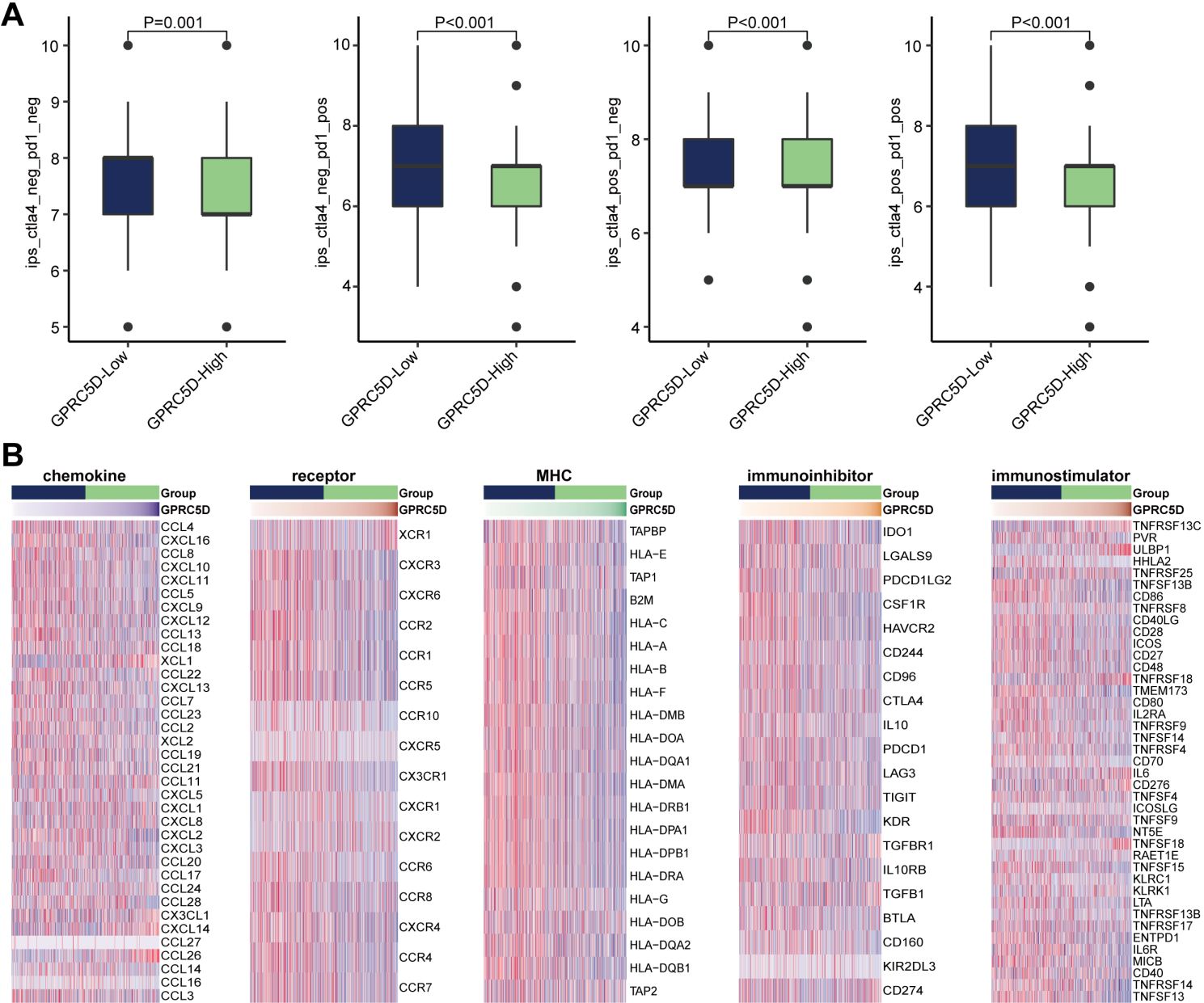
**

**Figure S2. Correlation between GPRC5D expression and (A) IPS and (B) immunomodulators.**

**
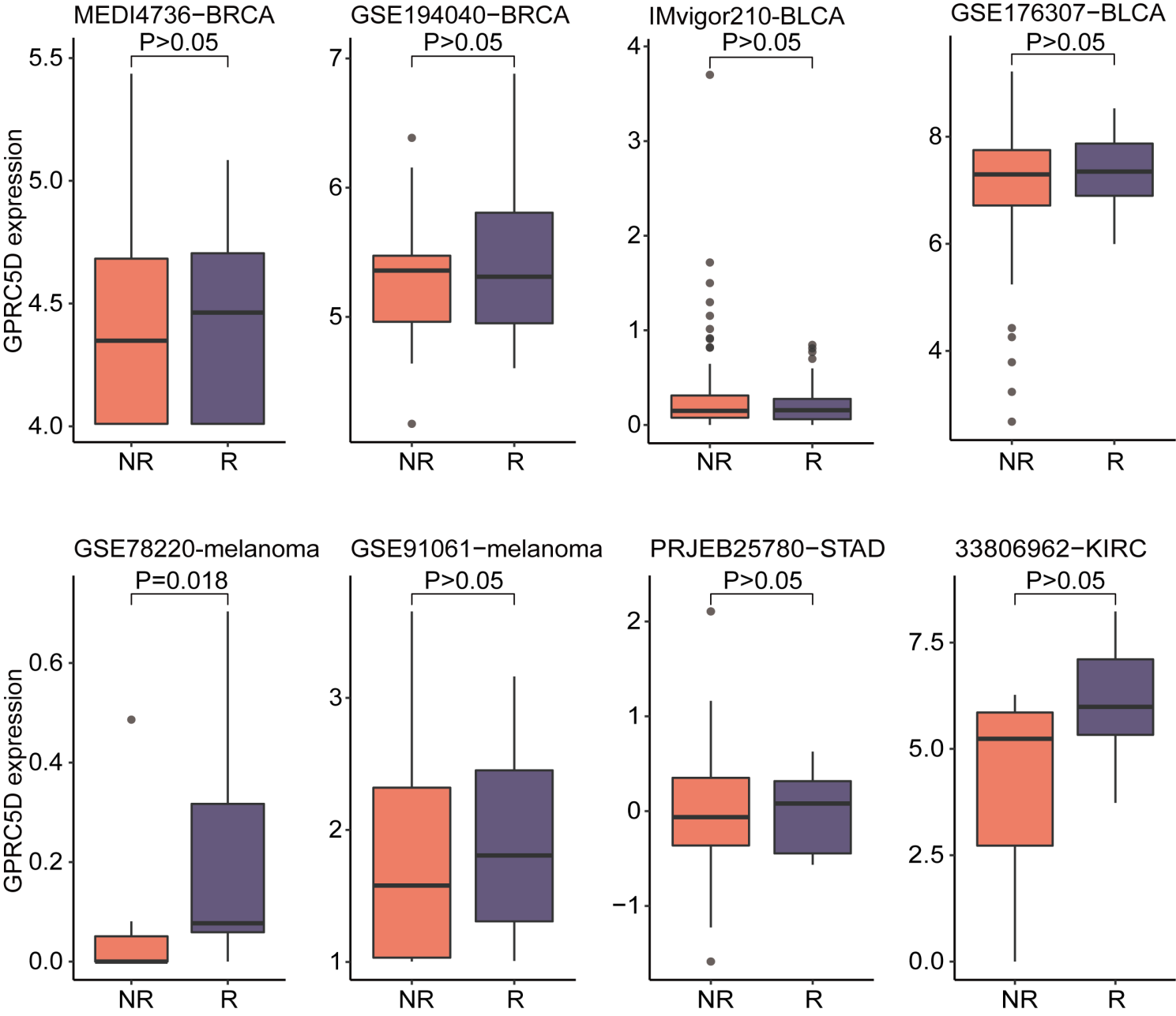
**

**Figure S3. Expression of GPRC5D in tumors from responders and non-responders in other cancer types**

**Additional Table**

**Table S1. The baseline clinic-pathological features of two cohorts.**

| **Clinic-pathological features** | **First cohort** | | **Second cohort** | |
| --- | --- | --- | --- | --- |
| Gender |  |  |  |  |
| Male | 31 | 64.58% | 26 | 83.87% |
| Female | 17 | 35.42% | 5 | 16.13% |
| Age |  |  |  |  |
| ≤60 | 21 | 43.75% | 7 | 22.58% |
| >60 | 27 | 56.25% | 24 | 77.42% |
| Pathological type |  |  |  |  |
| Adenocarcinoma | 37 | 77.08% | 21 | 67.74% |
| Non-adenocarcinoma | 11 | 22.92% | 10 | 32.26% |
| Differentiation |  |  |  |  |
| High/Medium | 22 | 45.83% | 12 | 38.71% |
| Poor | 26 | 54.17% | 19 | 61.29% |
| Therapeutic response |  |  |  |  |
| CR+PR | - | - | 7 | 22.58% |
| SD | - | - | 6 | 19.35% |
| PD | - | - | 18 | 58.06% |
